# The effects of methylphenidate on cognitive performance of healthy male rats

**DOI:** 10.3389/fnins.2013.00097

**Published:** 2013-06-13

**Authors:** Claire L. Rostron, Elise Kaplan, Victoria Gaeta, Rachel Nigriello, Eleanor J. Dommett

**Affiliations:** Brain and Behavioural Sciences, Biomedical Research Network, Department of Life, Health and Chemical Sciences, The Open UniversityMilton Keynes, UK

**Keywords:** cognitive enhancement, radial maze, state-dependent effects, hippocampus, prefrontal cortex, nucleus accumbens, dopamine

## Abstract

We aimed to investigate the effects of methylphenidate (MPH) in healthy rats on two distinct radial maze tasks which rely on brain structures and neurotransmitters known to be affected by MPH: the Random Foraging Non-Delay Task (RFNDT) and the Delayed Spatial Win Shift Task (DSWT). Hooded Lister rats were trained to complete either the RFNDT or the DSWT having received oral treatment of either a vehicle or MPH (3.0 mg/kg and 5.0 mg/kg for RFNDT, 3.0 mg/kg for DSWT). We found no effect of MPH on the RFNDT relative to the control group. However, those treated with 5.0 mg/kg MPH did take significantly longer to reach criterion performance than those treated with the 3.0 mg/kg MPH, suggesting some doses of MPH can have detrimental effects. For the DSWT, if MPH was present in both phases, performance did not differ from when it was absent in both phases. However, when present in only one phase there was an increase in errors made, although this only reached significance for when MPH was present only in the test-phase. These data suggest that MPH may have detrimental effects on task performance and can result in state-dependent effects in healthy individuals.

## Introduction

The term “nootropic” was coined in 1964 to mean drugs capable of enhancing cognition. At the time it referred to a limited range of drugs that improved performance on simple learning and memory tasks in rodents. Almost 50 years later, “nootropic” has been replaced by “cognitive enhancer” and the focus is on drugs such as methylphenidate (MPH) and their effects in humans. MPH is commonly used to treat Attention Deficit Hyperactivity Disorder (ADHD) but there is increasing off-label use by healthy individuals seeking to boost cognition (Morris, 2008). It is reported that, on average, 7% of US university students are using cognitive enhancers, a figure that rises to 25% at some institutions (Sahakian and Morein-Zamir, 2007; Greely et al., 2008).

Despite this increasing use of MPH in healthy individuals, research into its effects has largely been conducted in those diagnosed with ADHD. Although the neurobiology of ADHD is not fully understood, it is generally accepted that the pathophysiology includes a role for both dopamine and noradrenalin (Arnsten, 2009; Del Campo et al., 2011). Both of these are affected by MPH. It would therefore be remiss to assume that the effects on cognition observed in this clinical population, with abnormalities in the very systems where the drug acts, would be mirrored in a healthy population. Of the limited research that has been conducted with a non-clinical population, improvements have been found in self-reported measures of attention (Koelega, 1993; Advokat et al., 2010), attention set shifting (Rogers et al., 1999), recall (Camp-Bruno and Herting, 1994), digit span (Agay et al., 2010), and spatial working memory (Elliott et al., 1997; Mehta et al., 2000). However, the exact effects of MPH depend on a number of factors. For example, it may be detrimental where performance is already established (Elliott et al., 1997) and where enhancement does occur it appears to be baseline dependent such that improvements are only found in those who perform poorly at baseline (Clatworthy et al., 2009).

Whilst research in non-clinical human populations has been limited, research in healthy animals has been more plentiful. This is largely due to the lack of an ideal animal model of ADHD which has resulted in the tendency to use healthy rat strains in MPH studies. However, findings in rats are inconsistent. Positive effects have been reported on a radial maze spatial memory task (Zhu et al., 2007), retention of contextual fear responding (Bethancourt et al., 2009), and long term potentiation and depression mechanisms (Dommett et al., 2008), but no effects have been reported on complex stimulus discrimination tasks (Galizio et al., 2009), or water maze performance (McFadyen et al., 2002). Furthermore, impairment of object recognition has also been observed (Chuhan and Taukulis, 2006).

In addition to the relative dearth of studies into the effects of MPH in healthy individuals, the majority of studies with humans have used acute drug administration (Camp-Bruno and Herting, 1994; Elliott et al., 1997; Rogers et al., 1999; Mehta et al., 2000; Agay et al., 2010), which may not reflect the pattern of use in healthy humans where the drug could be taken over a longer time, for example during revision and exam periods. Indeed, a recent review and meta-analysis of the effects of MPH and modafinil in healthy humans, encompassing all single, or double blind placebo controlled studies, revealed only two studies with repeated administration (Repantis et al., 2010). The first of these found no effects on any measures collected (memory, mood, or wakefulness) (Gilbert et al., 1973), whilst the second found only an increase in anxiety following MPH treatment (Gobbi et al., 2003). The use of repeated drug administration is more common in animal work with the majority of the studies outlined above using repeated administration (McFadyen et al., 2002; Zhu et al., 2007; Bethancourt et al., 2009; Galizio et al., 2009). Nevertheless, many of the studies using repeated administration in animals have administered the drug by injection which does not give comparable pharmacokinetics to the oral administration used in humans. In addition, many used doses that do not provide comparable blood plasma levels to those found in humans taking the drug (Kuczenski and Segal, 2002). Therefore, even where repeated administration has been used, relevance to human use is limited.

Examination of the neurochemical effects of orally administered MPH may provide useful insights into what kind of behavioral measures might be sensitive to MPH-induced changes. As stated above, MPH affects both dopamine and noradrenalin by acting as a reuptake blocker for both monoamines. Given the diffuse innervation of these monoamines fibers, the effects on the brain are likely to be widespread. However, three structures have been the focus of a number of studies using oral administration in the rat at doses relevant to humans: the nucleus accumbens (NAc), prefrontal cortex (PFC), and hippocampus. These studies have revealed that acute orally administered MPH increased dopamine in the NAc (Kuczenski and Segal, 2002; Berridge et al., 2006) and in the PFC (Berridge et al., 2006) and increased noradrenalin levels in the hippocampus (Kuczenski and Segal, 2002). In all cases increased neurotransmitter was found after 20 min and persisted for at least a further 20 min (Kuczenski and Segal, 2002; Berridge et al., 2006). Given this information, it seems probable that cognitive tasks reliant on these brain regions that can also be conducted within the 20 min time period during which MPH has increased neurotransmission, are likely to be sensitive to MPH.

The PFC-NAc-hippocampal circuitry has been implicated in two distinct types of spatial memory, which can be differentiated using a radial maze. Originally developed to test hippocampal-dependent memory (Olton and Papas, 1979), it is now clear there is a role for the prelimbic area of the PFC as well as the NAc (Seamans and Phillips, 1994; Seamans et al., 1995; Floresco et al., 1997) in radial maze performance. These two regions are strongly connected with the ventral CA1/subciculum region of the hippocampus (Groenewegen et al., 1987; Jay and Witter, 1991) and at a behavioral level this connectivity supports two distinct strategies for spatial foraging. The prelimbic PFC to hippocampus connection plays a role specifically in a delayed spatial win shift task on the radial maze (DSWT). This task has two phases separated by a delay. Successful foraging for 4/8 baited arms in the second phase is reliant on accurate memory of food location prior to the delay, as well as a shift strategy because food is located in the four arms that were not baited in the first phase (the exact location of baited arms changes every day). The PFC dependency occurs because rats begin the second phase with prior information about where to locate food reward and must recall this, actively updating their memory as they work through to complete this second phase. In contrast to the PFC, the NAc appears to be involved in successful performance of both the DSWT and a random foraging non-delay task (RFNDT), indicative of a general role in goal-directed motor behavior (Kelley and Stinus, 1985; Mogenson et al., 1993; Yin et al., 2008).

Therefore, given the known pharmacological effects of orally administered MPH on the three key regions involved in radial maze performance and knowledge of the specific radial maze task demands that depend on these structures, and the connections between them, we suggest these are the optimal task in which to investigate cognitive enhancing effects of MPH. As such we investigated the effects of orally administered MPH in healthy Hooded Lister rats by examining acquisition of the RFNDT and DSWT, hypothesizing that orally administered MPH would improve performance on both tasks in comparison to rats treated with a vehicle solution.

## Materials and methods

### Subjects

A total of fifty-four male Hooded Lister rats (Harlan, UK) aged 60 days at the start of testing were used. Rats were housed at a constant temperature of 21–23°C on a 12:12 h light/dark cycle in cages of three and placed on food restriction such that they received a daily amount of 18 g of lab chow per rat. This level ensured healthy growth but sufficient motivation to complete the tasks. Rats were weighed daily throughout the study. Behavioral testing took place during the light period and feeding occurred once all rats had been tested each day. All work was conducted in line with local ethical procedures and the Animals (Scientific Procedures) Act 1986.

### Apparatus

Behavioral training and testing was carried out on an elevated eight arm, gray plastic radial maze. There was a food cup positioned 1 cm from the far end of each arm and guillotine style doors were used to restrict access to the arms when necessary. The central platform was 45 cm in diameter and the arms were 68 cm long and 10 cm wide. The arms of the maze did not have full sides. Rather they had a 2 cm lip on all sides of arms which allowed maximum visibility of the animal and reward pellet by the experimenter. Where the arms connected to the central platform the height of the side increased to that of the guillotine doors (12 cm) over a length of 10 cm. This was necessary to prevent rats moving directly from one arm to another without travelling via the central platform. The maze was elevated 50 cm above the floor. Observations were directly recorded onto the PC using a specially written Python programme. There were some extra maze cues in the testing room (signs of black and white symbols and other equipment) but these remained constant throughout the study.

### Random foraging non-delay task (RFNDT)

A total of thirty-six rats were used for this task. They were split into two cohorts for testing because of the time-consuming nature of the task. The first cohort received either treatment with 3.0 mg/kg MPH (*N* = 9) or vehicle (*N* = 9), whilst the second cohort received either treatment with 5.0 mg/kg MPH (*N* = 9) or vehicle (*N* = 9). This approach was taken to avoid using a historical control group.

**Figure 1:**
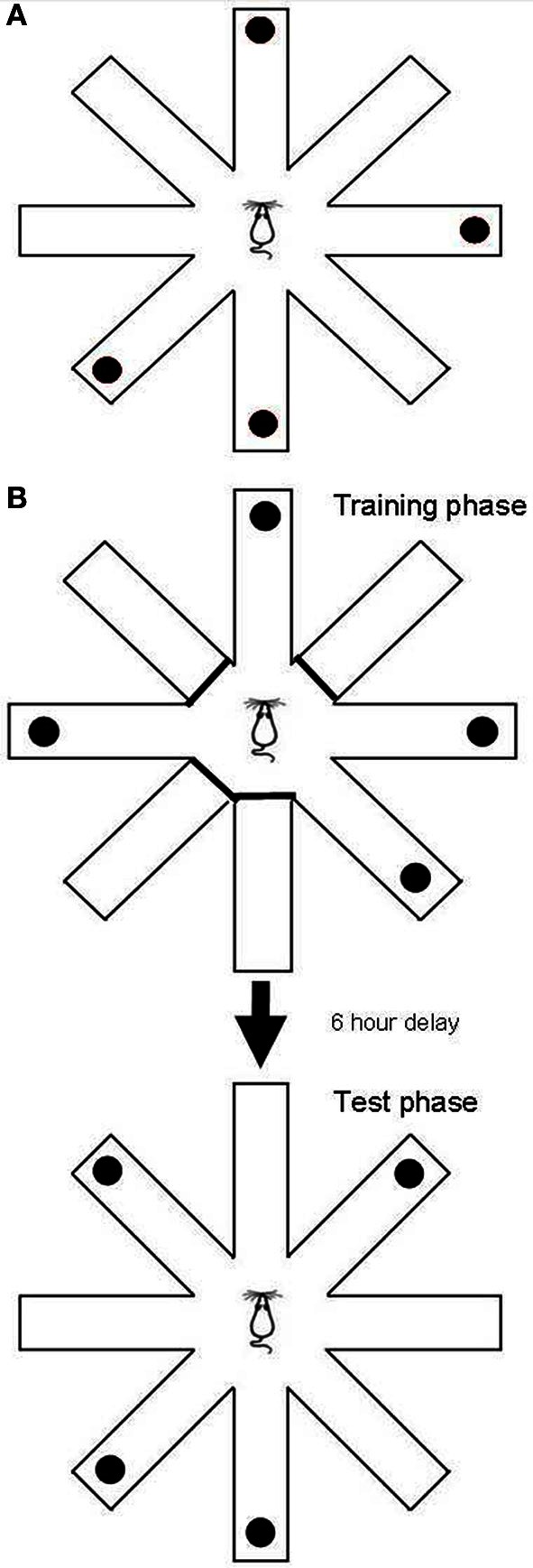
**(A)** The RFNDT required the rat to locate four pellets located in randomly chosen arms with all eight arms open. **(B)** The DSWT task had two phases: the training phase in which four randomly selected arms were open and baited, and the test phase, in which all arms were open and the four previously closed arms were baited.

The RFNDT has been described before (Keating and Winn, 2002) and is illustrated in Figure 1A. Prior to training on this task all rats were habituated to the experimenter and the maze. In addition, they were habituated to the reward pellets in the home cage. A single trial of the RFNDT required the rat to find four reward pellets from eight open arms. Pellets were raspberry-flavored 45 mg pellets (TestDiet 5TUM 18123372, Sandown Scientific, UK). Although these pellets were not odorless previous work has confirmed that this task does not rely on olfactory cues (Olton and Samuelson, 1976; Zoladek and Roberts, 1978; Olton and Collison, 1979). The pellets were placed in four randomly chosen arms on each trial using a random number table such that no more than two consecutive arms were baited or unbaited in a trial, and at least one arm was different from the previous day's trial. At the start of each trial the rat was placed on the central platform and given 10 min to find all four pellets. When all pellets had been eaten (or 10 min elapsed) the rat was removed from the maze and returned to its home cage. The maze was washed down with alcohol spray between each trial to remove olfactory cues from each rat. Arm entries were recorded once the animal had all four paws on the arm. Arm exits were recorded once the animal had all four paws on the central platform. Each rat underwent one trial per day. The dependent variables were recorded direct to a Python computer programme by an observer in the room who was blind to the drug condition. The dependent variables were: latency to first arm choice; mean arm choice time (defined as the total time on the maze divided by the number of choices made); number of unbaited entries; number of unbaited re-entries; number of baited entries; number of baited re-entries; and total number of re-entries. This last variable, “total number of re-entries,” was used for determining criterion performance which was defined as a rat collecting all four reward pellets with one or less re-entry error over 2 consecutive days. On an individual basis this was applied to determine “expertise” on the task. Testing was continued for all rats until the final animal had reached criterion. The number of pellets eaten was also recorded throughout but very early on in testing this consistently reached the maximum and therefore came to equal the number of baited arm entries.

All data were deemed suitable for analysis with parametric tests following normality checks using the Kolmogorov-Smirnov test and, where necessary, corrections for violations of homogeneity of variance were applied (Greenhouse-Geisser). Additionally, analysis revealed that the control groups from each cohort did not differ significantly from each other on any measures and therefore the data from the two cohorts were combined into a single, more powerful analysis (*N* = 9 for each drug dose and *N* = 18 for vehicle). The number of trials needed to reach criterion were analyzed using a One Way ANOVA. All other variables were analyzed with a 1 within (TRIAL), 1 between (TREATMENT) ANOVA.

Following completion on the RFNDT all animals underwent a single 75 min session of locomotor monitoring using automated Activity Monitoring Chambers (Med-Associates). By this point animals tested in the first batch (control and low dose) were 165 days old and those in the second (control and high dose) were 126 days old. This allowed at least 1 month between the final test day on the maze and locomotor testing. This was necessary in order to determine whether drug treatment significantly altered locomotor activity in a way that could explain or confound RFNDT performance. Animals were habituated to the chambers for 15 min on 2 consecutive days before monitoring took place. For data collection, rats received treatment as per the conditions of the RFNDT (i.e., the same treatment was provided such that overall there were *N* = 9 3.0 mg/kg, *N* = 9 5.0 mg/kg, and *N* = 18 Vehicle). Data were binned into 5 min blocks and collected for the full 75 min. This duration was selected as it has previously been shown to be sufficient for neurotransmitter levels to return to pre-MPH levels (Kuczenski and Segal, 2002). Four behavioral measures were recorded: ambulatory counts (number of beam breaks), ambulatory distance (horizontal distance travelled), average velocity, and stereotypic activity (any partial-body movements that occur within a defined space, such as head-weaving, or scratching movements but may also include grooming). As with the RFNDT data, Kolmogorov-Smirnov tests were used to confirm normality of distribution, and the two cohorts were collapsed after tests revealed no significant differences between the two control groups on any measure, allowing combined analyses. Data were analyzed using a 1 within (BLOCK), 1 between (TREATMENT) ANOVA.

### Delayed spatial win shift task (DSWT)

The remaining 18 rats were used for the DSWT. This task has also been described in detail before (Keating and Winn, 2002; Taylor et al., 2003, 2004) and is illustrated in Figure 1B. As with the RFNDT each rat underwent one trial per day. A single trial of the DSWT consists of two phases. The first phase (training phase) requires the animal to find four food rewards from four open arms, with the remaining four arms blocked off. Baited arms are randomly chosen each day and no more than two consecutive arms are allowed. In the second post-delay phase (test phase), the animal is required to find four reward pellets from eight open arms. In this phase the pellets are placed in the arms that did not contain pellets in the training phase. The animal must therefore “shift” to “win” the task and find the four pellets in the test phase. All animals were trained to a criterion of completing both the training and test phases with no more than five total arm entries per phase. Following acquisition of criterion with a 5 min delay between phases the delay was then increased to 30 min, then 1 h, then 3 h, and finally 6 h. A total of 80 trials were completed for this task across all delay periods. A 6 h delay period has previously been shown to significantly increase error rates (Phillips et al., 2004) therefore providing a suitable platform to investigate any MPH induced improvements in DSWT performance. This delay (6 h) also far exceeds the time taken for oral MPH to washout in rats (approximately 2 h) (Patrick et al., 1984) and for any changes in transmitter levels induced by the drug to return to baseline levels (Kuczenski and Segal, 2002; Berridge et al., 2006). Therefore, any effects of MPH administered in the training phase would have returned to baseline levels before the test phase. Thus, drug effects relevant to each stage could therefore be neatly dissociated. Prior to beginning the task with a 6 h delay rats were randomly allocated into four groups: those that received a vehicle in both phases (*N* = 5); those that received MPH in both phases (*N* = 5); those that received vehicle in the training phase and MPH in the test phase (*N* = 4); and those that received MPH in the training phase and vehicle in the test phase (*N* = 4). Therefore, it is important to note that rats only received MPH or vehicle once they progressed to the 6 h delay and not at any of the previous delays. Unlike the RFNDT, rat numbers allowed all rats to be tested as a single cohort.

Arm entries and exits were as defined in the RFNDT. Four dependent variables were measured in both phases of the DSWT: baited arm entry, baited arm re-entry, latency to reach the first arm choice, and mean arm choice time (time to complete the phase/number of choices made). In the test phase a number of additional measures were collected. Firstly, unbaited arm entries were recorded. These constitute entries into any of the training phase arms which are, by design, unbaited in the test phase. Such errors are referred to as “across phase” errors and has a ceiling score of four. Secondly, re-entry into unbaited arms was measured. Finally the total re-entry into any arm previously visited in the test phase, termed “within phase” errors was measured. As with the first task all data were deemed suitable for analysis with parametric tests following normality checks using the Kolmogorov-Smirnov test and where necessary corrections for violations of homogeneity of variance were applied (Greenhouse-Geisser). All analyses were therefore conducted using a 1 within (TRIAL), 1 between (TREATMENT) ANOVA.

### Drug treatment

Animals were trained to receive oral administration of apple juice for 5 days prior to starting any behavioral training. This method removes the ethical and welfare concerns of repeated gavage and closely replicates the method of administration in humans (Wheeler et al., 2007). In addition, once trained the animals easily receive the juice even after long periods without administration. This allows habituation to the administration technique to take place prior to training on a behavior task. A stock solution of MPH hydrochloride (Sigma, UK) was made in distilled water and frozen at −20°C until use. Immediately prior to use it was defrosted and diluted into apple juice to give either 3.0 mg/kg or 5.0 mg/kg dose. The vehicle solution consisted of the same volume of distilled water, also previously frozen, diluted into apple juice immediately prior to use. Previous research has shown that MPH has therapeutic efficacy in treating individuals with ADHD when blood plasma levels are in the range of 8–40 ng/ml (Swanson and Volkow, 2001). Given one of the main sources for MPH as a cognitive enhancer is likely to be the ADHD prescribed medication it is appropriate to use similar doses in the present study. It has also been demonstrated that this level can be achieved in rats using the same method of oral administration in apple juice employed here with doses between 1 mg/kg (9.56 ng/ml) and 5 mg/kg (36.61 ng/ml) (Wheeler et al., 2007). These levels were reached 15 min after administration, allowing time for the blood plasma levels to peak (Patrick et al., 1984), and around 5 min before effects on neurotransmitter levels have been shown to peak after oral administration (Kuczenski and Segal, 2002; Berridge et al., 2006). Although doses as low as 1 mg/kg have been shown to be therapeutically relevant when administered orally there is some inconsistency in the effects on dopamine and noradrenalin levels at the lower levels of the therapeutic range. For example, previous research using oral administration, albeit by gavage, has found a dose of 2 mg/kg increases dopamine levels in the PFC and NAc (Berridge et al., 2006), whilst others found that 2.5 mg/kg does not increase dopamine levels in the NAc (PFC not tested) but 5.0 mg/kg does (Kuczenski and Segal, 2002). For noradrenalin, previous research has revealed increases in hippocampal noradrenalin with orally administered doses as low as 1.0 mg/kg (Kuczenski and Segal, 2002), whilst intraperitoneally administered MPH was shown to increase noradrenalin in the PFC (Berridge et al., 2006) at doses corresponding to mid and high therapeutic doses. Due to the inconsistent previous findings for lower doses of MPH we opted for a “low dose” of 3.0 mg/kg which would, with this route of administration, represent mid therapeutic levels and a “high dose” of 5.0 mg/kg, corresponding to the higher end of the range. Administration was 20 min prior to placement on the maze or in activity monitoring chambers based on previous research demonstrating that this period was sufficient for orally administered drug to have significant effects on noradrenalin and dopamine levels (Kuczenski and Segal, 2002; Berridge et al., 2006). All behavioral testing was conducted blind to the substance administered.

## Results

### Low and high doses of MPH had significantly different effects on the time taken to reach criterion performance on the RFNDT but did not affect locomotor activity

**Figure 2:**
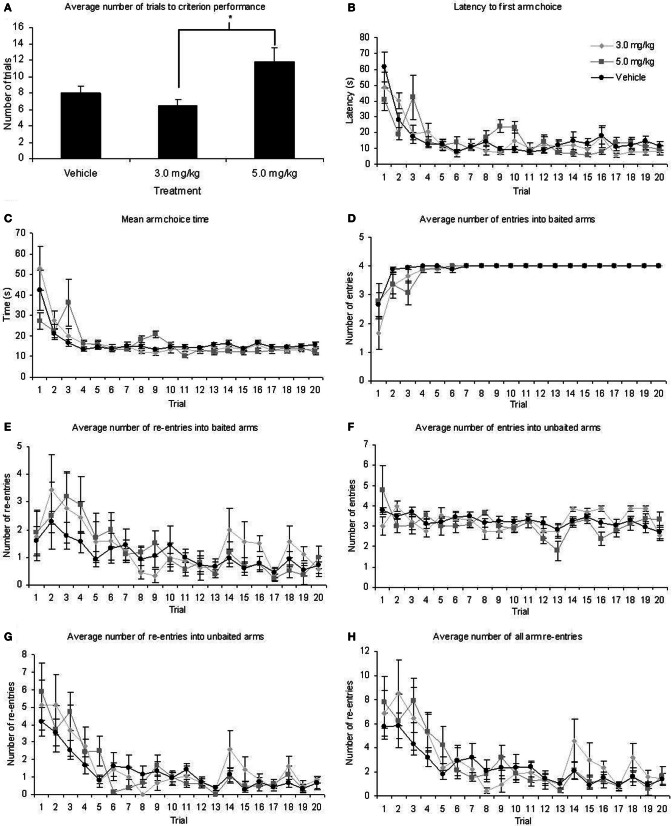
**(A)** Rats treated with 5.0 mg/kg MPH took significantly longer to reach criterion on the RFNDT than those treated with 3.0 mg/kg although neither differed significantly from the vehicle-treated group. However, there were no significant main effects of treatment for any other measure of radial maze performance, ^*^p < 0.05: **(B)** latency to first arm choice **(C)** mean arm choice time **(D)** baited arm entry **(E)** baited arm re-entry **(F)** unbaited arm entry **(G)** unbaited arm re-entry and **(H)** total re-entries made. All graphs show mean ± SEM.

All rats reached criterion performance (an average of 1 or less re-entry errors on two consecutive days) within 20 days of testing, indicating they had attained a level of expertise on the task. One Way ANOVA revealed there was a significant difference in the number of days taken to reach criterion performance between the three treatment conditions [*F*_(2, 35)_ = 4.78; *p* = 0.015]. Figure 2A shows that the fastest group to reach criterion were those treated with 3.0 mg/kg MPH (mean ± SEM, 6.44 ± 0.69 days), whilst the slowest were those treated with 5.0 mg/kg MPH (11.78 ± 1.73 days). *Post-hoc* Tukey tests revealed that the rats treated with 3.0 mg/kg MPH were not significantly different from those treated with the vehicle (*p* < 0.582) but were significantly quicker to reach criterion performance than those treated with 5.0 mg/kg MPH (*p* = 0.015). The group treated with 5.0 mg/kg showed a trend toward being significantly slower than those treated with the vehicle (*p* = 0.053) who took 8 ± 0.87 days to reach criterion.

All remaining data were analyzed with a 1 within (TRIAL), 1 between (TREATMENT) ANOVA (Table 1; Figures 2B–H). There were significant main effects of TRIAL for all dependent variables. These main effects were as expected as performance improves with number of trials. There were no significant main effects of TREATMENT on any of the measures taken. In the majority of cases there was no significant TREATMENT × TRIAL interactions. However, significant interactions were found for latency and unbaited arm entry. For latency, *post-hoc* ANOVA and Tukey tests revealed significant differences between the three treatment groups on trials 2, 3, 9, and 10. For trial 2, those treated with 3.0 mg/kg MPH had a significantly higher latency than those in the 5.0 mg/kg group. However, in the remaining trials where significant differences were found the group given 5.0 mg/kg MPH had a significantly higher latency than the vehicle treated group (Trials 3, 9, and 10) and the 3.0mg/kg treated group (Trial 9). It is likely the differences seen in these trials resulted in the interaction effects seen. This is further supported with a 1 within 1 between ANOVA excluding these trials resulting in a non-significant interaction effect. For unbaited re-entry, the same *post-hoc* tests reveal significant differences between the two drug treatment conditions on trials 2 and 16 and a significant difference between the 3.0 mg/kg treatment group and the vehicle group on trial 19. In all three cases the 3.0 mg/kg MPH group had greater re-entry errors. It is likely these trials resulted in the interaction effects seen. This is further supported with a 1 within 1 between ANOVA excluding these trials resulting in a non-significant interaction effect. Despite these significant interactions, the greatest differences seen in estimated marginal means equated to less than 2 arm re-entries and 25 s on the maze, indicating these interactions are likely to be a result of individual differences in performance.

**Table 1:** **Results of 1 within (TRIAL), 1 between (TREATMENT) ANOVA on the RFNDT**.

**Measure**	**Main effect of treatment**	**Main effect of trial**	**Interaction effect**
Latency to first arm choice	*F*_(2, 33)_ = 0.09	*F*_(4.89, 161.29)_ = 16.94^**^	*F*_(9.78, 161.29)_ = 2.40^*^
Mean arm choice time	*F*_(2, 33)_ = 0.02	*F*_(2.09, 69.10)_ = 13.39^**^	*F*_(4.19, 69.10)_ = 1.74
Baited entry	*F*_(2, 33)_ = 2.32	*F*_(2.29, 75.51)_ = 17.49^**^	*F*_(4.58, 75.51)_ = 1.57
Baited re-entry	*F*_(2, 32)_ = 1.26	*F*_(8.06, 257.91)_ = 5.14^**^	*F*_(16.12, 257.91)_ = 0.67
Unbaited entry	*F*_(2, 33)_ = 1.14	*F*_(9.34, 308.33)_ = 2.97^*^	*F*_(18.67, 308.33)_ = 1.73^*^
Unbaited re-entry	*F*_(2, 33)_ = 0.50	*F*_(6.43, 212.15)_ = 13.86^**^	*F*_(12.86, 212.15)_ = 1.02
Total re-entry	*F*_(2, 32)_ = 1.02	*F*_(7.73, 247.46)_ = 10.72^**^	*F*_(15.47, 247.48)_ = 0.90

*There were no significant main effects of TREATMENT. There were significant effects of TRIAL as the animals' performance improved with increased trials. There were two significant interaction effects for latency and unbaited arm entry*.

* *p < 0.05*,

** p < 0.001.

Previous research (Zhu et al., 2007) found effects of a similar dose of orally administered MPH when only the early trials were examined. As such we repeated the ANOVAs outlined above restricting the trials to 1–5. We found no significant main effects of TREATMENT for any of the measures indicating that even in the first 5 days when the most learning occurs, MPH had no effect on the different measures of maze activity. These restricted ANOVAs showed similar significant main effects of TRIAL as the full ANOVA for latency, mean arm choice time, baited entry, unbaited re-entry, and total-re-entry. However, there was no significant main effect of TRIAL on baited re-entry and unbaited entry when this restricted period was considered. There were no significant interactions with this restricted period for all measures except latency which showed a significant interaction, as in the main ANOVA. *Post-hoc* tests reveal that this is likely to be due to the significant group differences on trials 2 and 3 that have been previously described above.

**Figure 3:**
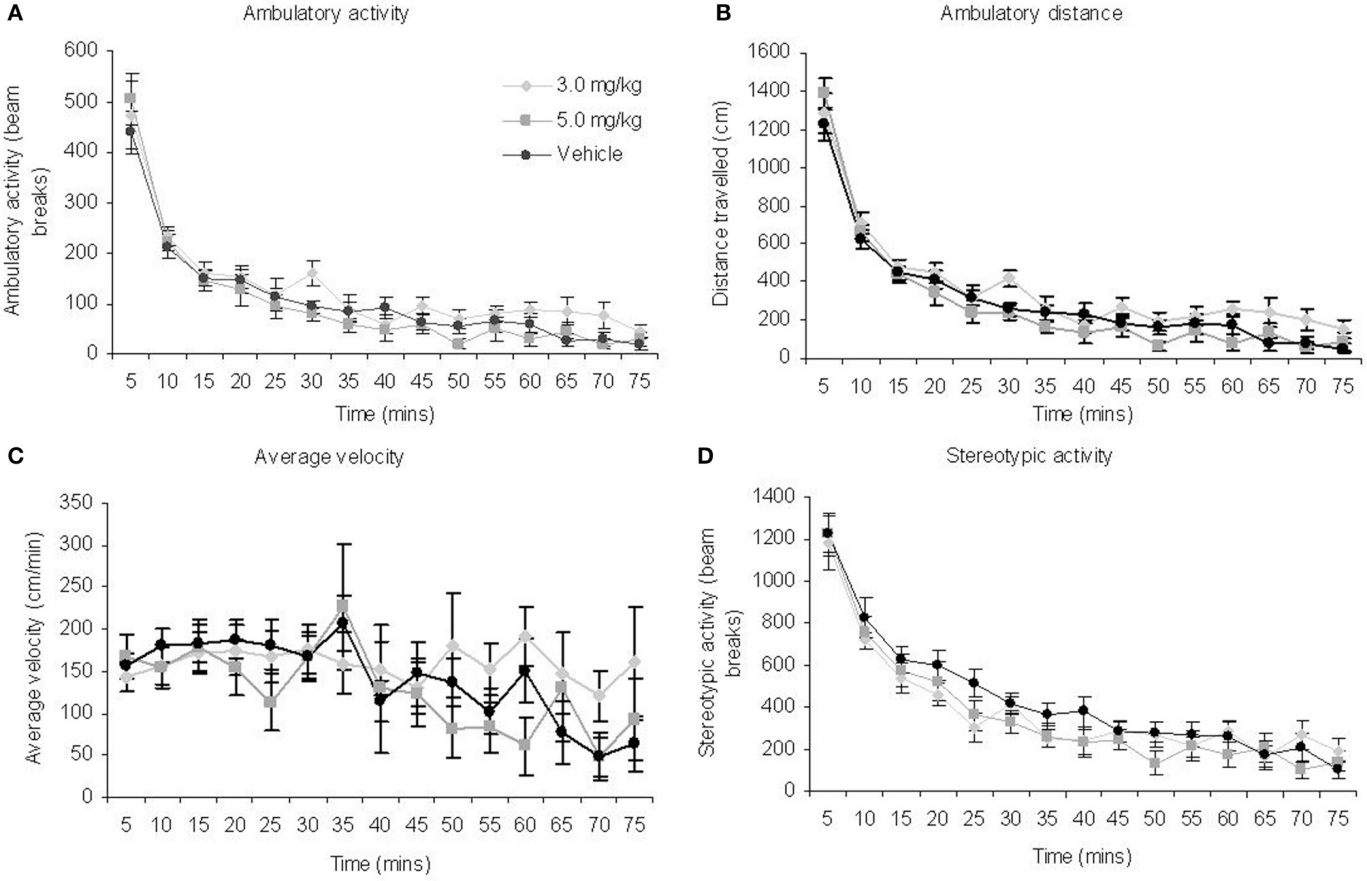
**There were no effects of MPH on measures of locomotor activity (A) ambulatory activity (B) ambulatory distance (C) average velocity (D) stereotypic activity for the animals that completed the RFNDT**. Note that stereotypic activity would also have captured normal grooming behavior which may explain the initially high levels. All graphs show mean ± SEM.

Activity monitoring of the three groups revealed significant effects of BLOCK for each measure (Table 2; Figures 3A–D). This is expected for locomotor activity as the rats reduce spontaneous behaviour with duration in the chamber. There were no significant main effects of TREATMENT or BLOCK × TREATMENT interactions for any of the measures. Given the rats spent only around 5 min on the radial maze during the RFNDT, we compared also the activity levels in the first 5 min block for all measures across treatment conditions. There were no significant differences for any of the measures: ambulatory activity [*F*_(2, 35)_ = 0.44; *p* > 0.05]; ambulatory distance [*F*_(2, 35)_ = 0.86; *p* > 0.05]; average velocity [*F*_(2, 35)_ = 0.29; *p* > 0.05]; and stereotypic activity [*F*_(2, 35)_ = 0.05; *p* > 0.05].

**Table 2:** **Results of 1 within (BLOCK), 1 between (TREATMENT) ANOVA on the locomotor activity**.

**Measure**	**Main effect of treatment**	**Main effect of block**	**Interaction effect**
Ambulatory counts	*F*_(2, 33)_ = 0.79	*F*_(4.41, 145.51)_ = 101.69^**^	*F*_(8.82, 145.51)_ = 0.94
Ambulatory distance	*F*_(2, 33)_ = 1.51	*F*_(7.25, 239.30)_ = 152.03^**^	*F*_(14.50, 239.30)_ = 1.26
Average velocity	*F*_(2, 33)_ = 0.48	*F*_(6.97, 230.14)_ = 3.17^*^	*F*_(3.95, 230.14)_ = 0.95
Stereotypic activity	*F*_(2, 33)_ = 0.56	*F*_(4.87, 160.76)_ = 85.89^**^	*F*_(9.74, 160.76)_ = 0.99

*There were no significant main effects of TREATMENT. There were significant effects of BLOCK as the animals' activity decreased with duration in the chamber. There were no significant interaction effects*.

* *p < 0.05*,

** p < 0.001.

### Low dose methylphenidate may result in a state-dependent effect on DWST

**Figure 4:**
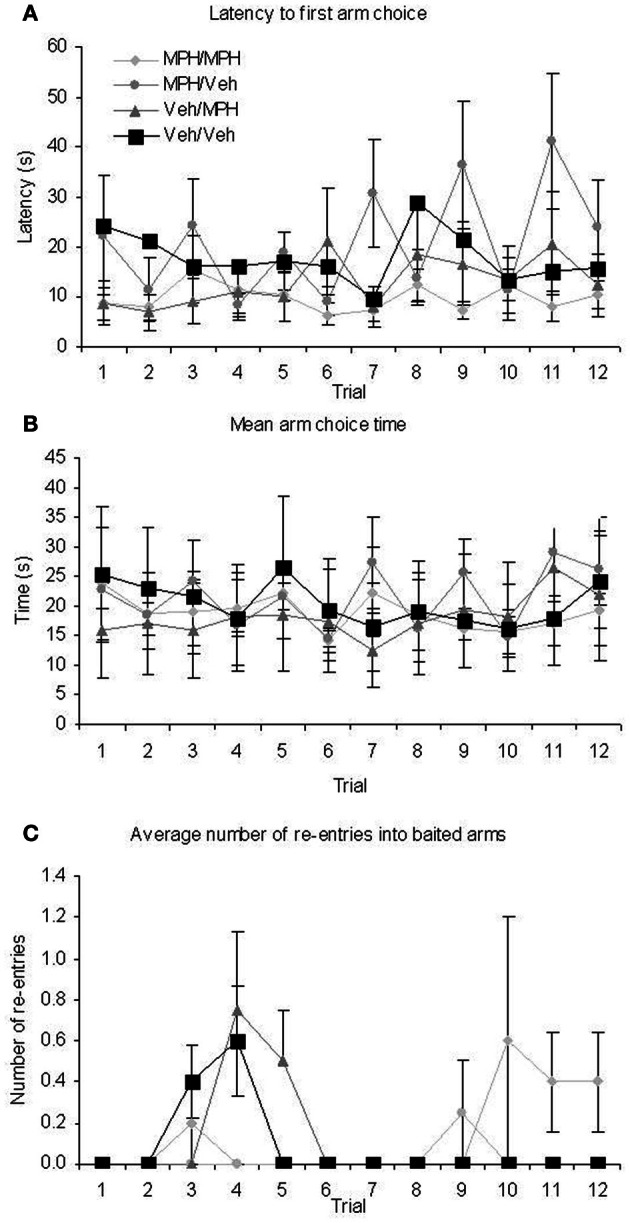
**Performance during the training phase on the 3 h DWST over the 12 trials run at this delay demonstrate that there were pre-existing group differences for the different drug treatment conditions used at the subsequent 6 h delay for (A) latency (B) mean arm choice time (C) re-entry in to baited arms**. All graphs show mean ± SEM.

Given the high dose had shown a trend toward impairing performance in the RFNDT and we were most interested in enhancement of performance in this study, we only used the lower dose for the DSWT. In order to be certain that any effects on performance at the 6 h delay were due to the administration of MPH rather than differences in group performance prior to this delay and drug administration, we examined performance on the training and test phases of the 3 h DSWT according to the treatment grouping used for the 6 h DSWT. All data were analyzed with a 1 within (TRIAL), 1 between (FUTURE TREATMENT) ANOVA for both the training and the test phases on this task. For the training phase all animals entered all four baited arms on every trial and therefore performance was identical across groups and trials for baited arm entry. For the remaining dependent variables, there was no main effect of TRIAL (Table 3, Figures 4A–C) or FUTURE TREATMENT on any measure. Finally, there were no TRIAL × TREATMENT interaction effects for baited re-entry or mean arm choice time. However, there was a significant interaction for latency. *Post-hoc* ANOVA and tukey tests revealed that there was a significant difference between the MPH/MPH and MPH/VEH group on trial 7 and trial 11 with the latter having a significantly longer latency in both cases (*p* < 0.05). It is likely that these differences resulted in the significant interaction effect, a suggestion supported by the lack of interaction seen when these trials were removed from the main analysis.

**Table 3:** **Results of 1 within (TRIAL), 1 between (FUTURE TREATMENT) ANOVA on the DSWT using a 3 h delay demonstrate that there were some differences between the four treatment groups used for the 6 h delay prior to any administration**.

	**Main effect of future treatment**	**Main effect of trial**	**Interaction effect**
**TRAINING PHASE MEASURE**			
Latency to first arm choice	*F*_(3, 14)_ = 1.25	*F*_(4.52, 63.31)_ = 1.81	*F*_(13.57, 63.31)_ = 1.96^*^
Mean arm choice time	*F*_(3, 14)_ = 0.47	*F*_(2.80, 39.15)_ = 1.30	*F*_(8.39, 39.15)_ = 0.72
Baited re-entry	*F*_(3, 14)_ = 0.49	*F*_(2.88, 39.51)_ = 1.39	*F*_(8.47, 39.51)_ = 1.32
**TESTING PHASE MEASURE**			
Latency to first arm choice	*F*_(3, 14)_ = 1.75	*F*_(4.46, 62.44)_ = 3.70^*^	*F*_(13.38, 62.44)_ = 1.82
Mean arm choice time	*F*_(3, 14)_ = 2.45	*F*_(4.98, 69.76)_ = 4.03^**^	*F*_(14.95, 69.76)_ = 1.14
Baited entry	*F*_(3, 14)_ = 1.21	*F*_(1, 14)_ = 1.296	*F*_(3, 14)_ = 1.21
Baited re-entry	*F*_(3, 14)_ = 5.56^*^	*F*_(1.94, 27.18)_ = 1.86	*F*_(5.82, 27.18)_ = 1.07
Unbaited re-entry	*F*_(3, 14)_ = 5.08^*^	*F*_(1.45, 20.28)_ = 1.62	*F*_(4.35, 20.28)_ = 1.52
Across phase errors	*F*_(3, 14)_ = 0.56	*F*_(11, 154)_ = 1.52	*F*_(33, 154)_ = 0.82
Within phase errors	*F*_(3, 14)_ = 8.30^*^	*F*_(2.05, 28.63)_ = 2.38	*F*_(6.14, 28.63)_ = 1.03
Total arm visits	*F*_(3, 14)_ = 4.94^*^	*F*_(4.38, 61.29)_ = 1.79	*F*_(13.13, 61.29)_ = 0.69

* *p < 0.05*,

** p < 0.001.

**Figure 5:**
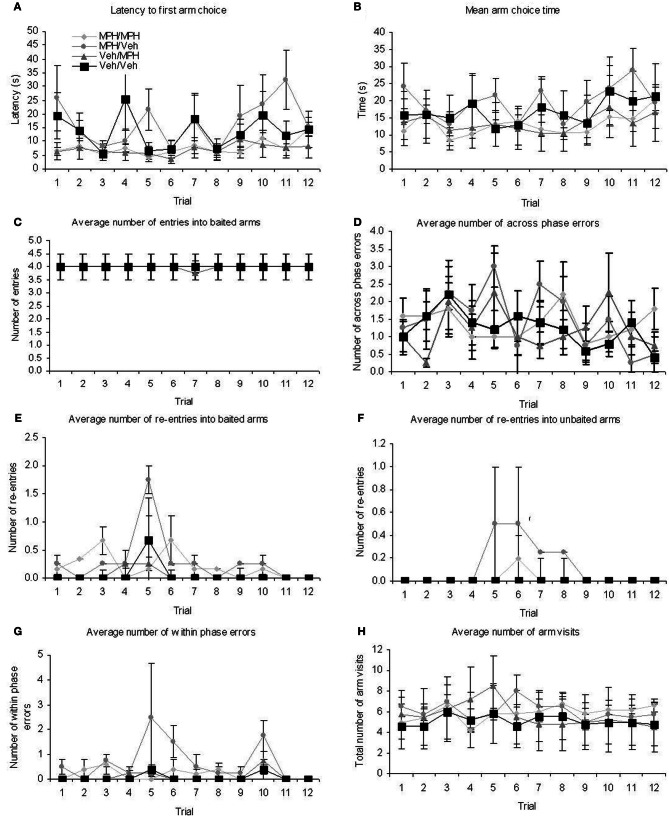
**Performance during the test phase on the 3 h DWST over the 12 trials run at this delay revealed that there were some pre-existing group differences for the treatment groups selected for the 6 h delay. (A)** latency **(B)** mean arm choice time **(C)** baited arm entry **(D)** across phase errors data did not show pre-existing differences. By contrast **(E)** baited arm re-entry **(F)** unbaited arm re-entry **(G)** within phase errors and **(H)** total number of arm visits data did show pre-existing differences. Prior to any drug treatment animals allocated to the MPH/Veh group made more within phase errors and re-entries into unbaited arms than the other three groups. These animals also showed greater baited re-entry in comparison to the Veh/MPH and the Veh/Veh group and more arm visits overall than the Veh/Veh group. These data demonstrate that prior to any drug treatment the rats subsequently allocated to the MPH/Veh group made more errors in the test phase. All graphs show mean ± SEM.

For the 3 h DSWT test phase there were main effects of TRIAL on latency and mean arm choice time (Table 3, Figure 5) and main effects of FUTURE TREATMENT on baited re-entry, unbaited re-entry, within phase errors, and total arm visits. For baited re-entry *post-hoc* Tukey tests show that prior to any drug administration, the animals allocated to the MPH/Veh group had significantly more re-entry errors than the Veh/MPH group (*p* = 0.019) and the Veh/Veh group (*p* = 0.011). Similarly, for unbaited re-entry, the animals allocated to the MPH/Veh group had significantly more unbaited re-entries than those in all other groups (MPH/MPH *p* = 0.024; Veh/MPH *p* = 0.033; Veh/Veh *p* = 0.024). For within phase errors *post-hoc* Tukey tests indicated that prior to any treatment the animals allocated to the MPH/Veh group made significantly more within phase errors than the Veh/Veh group (*p* = 0.002); MPH/MPH group (*p* = 0.014) and the Veh/MPH group (*p* = 0.006). Finally for the total arm visits, animals allocated to the MPH/Veh group showed significantly more arm entries than those allocated to the Veh/Veh groups (*p* = 0.011). These data indicate that prior to any drug treatment the rats subsequently allocated to the MPH/Veh group made more errors in the test phase. There were no significant TRIAL × FUTURE TREATMENT interactions.

Given the presence of significant main effects and interactions prior to actual drug treatment in the 6 h delay, we conducted an Analysis of Covariance for the 6 h DSWT with the individual mean score for each measure at the appropriate phase for the 3 h delay as a fixed covariate. A fully factorial ANCOVA includes all interaction terms between the covariate, within- and between-factors. Main effects of the within factor (TRIAL) are independent of the performance at the 3 h delay and therefore, pure repeated-measures effects are reported from an analysis that excludes the covariate, and so degrees of freedom differ for pure within effects and between effects or interactions.

**Figure 6:**
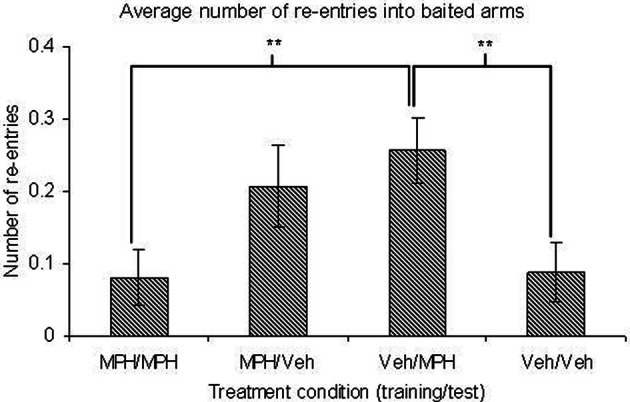
**With performance on the 3 h DSWT as a fixed covariate, an ANCOVA showed there are no significant differences in the number of baited re-entries between groups given methylphenidate (MPH) or vehicle (Veh) in both phases on the 6 h delay**. However, giving methylphenidate in just one phase increases the number of errors. When methylphenidate is given only in the test phase, this increase is significantly different from groups given the same treatment in both phases. All graphs show mean ± SEM. ^**^p = 0.01.

During the training phase there was no significant main effect of TRIAL or TREATMENT on any of the dependent variables (Table 4). Similarly there were no significant interaction effects between these variables. During the test phase there were no main effects of TRIAL on any dependent variable. For the majority of variables there was no significant main effect of TREATMENT. However, there was a significant effect of TREATMENT on baited re-entry errors (Figure 6). Contrast analysis revealed that there were no significant differences between the group given the vehicle in both phases and the group given MPH in both phases or just given MPH in training phase. However, if MPH was given in just the test phase, there were significantly more re-entry errors into baited arms relative to the group given solely the vehicle (*p* = 0.01) or solely MPH (*p* = 0.01) (Figure 6). There was also a trend toward a significant main effect of TREATMENT for across phase errors. Contrast analysis showed that this was likely due to the number of across phase errors made when MPH was present in both phases or just the test phase being higher than when it was present in only the training phase (*p* < 0.05).

**Table 4:** **Results of 1 within (TRIAL), 1 between (TREATMENT) ANCOVA for the 6 h DSWT, with performance at the 3 h delay as a fixed covariate**.

	**Main effect of treatment**	**Main effect of trial**	**Interaction effect**
**TRAINING PHASE MEASURE**			
Latency to first arm choice	*F*_(1, 15)_ = 0.32	*F*_(1.45, 23.18)_ = 0.64	*F*_(1.47, 29.17)_ = 0.91
Mean arm choice time	*F*_(1, 15)_ = 0.03	*F*_(3.54, 56.67)_ = 1.03	*F*_(4.04, 60.61)_ = 1.30
Baited entry	*F*_(1, 15)_ = 0	*F*_(1.98, 31.61)_ = 0.89	*F*_(1.98, 31.61)_ = 1.11
Baited re-entry	*F*_(1, 15)_ = 1.38	*F*_(3.70, 59.17)_ = 1.17	*F*_(3.67, 54.99)_ = 0.84
**TESTING PHASE MEASURE**			
Latency to first arm choice	*F*_(3, 12)_ = 0.53	*F*_(2.42, 31.39)_ = 2.30	*F*_(7.16, 28.65)_ = 1.21
Mean arm choice time	*F*_(3, 12)_ = 0.81	*F*_(4.49, 58.34)_ = 1.268	*F*_(12.50, 49.99)_ = 0.95
Baited entry	*F*_(3, 13)_ = 0	*F*_(1, 14)_ = 1.30	*F*_(1, 14)_ = 0
Baited re-entry	*F*_(3, 13)_ = 4.66^*^	*F*_(3.55, 49.77)_ = 3.50	*F*_(9.74, 42.22)_ = 3.70^**^
Unbaited re-entry	*F*_(3, 13)_ = 1.84	*F*_(2.78, 38.85)_ = 2.24	*F*_(8.39, 36.36)_ = 1.29
Across phase errors	*F*_(3, 13)_ = 3.05^α^	*F*_(4.68, 65.54)_ = 1.15	*F*_(13.96, 60.51)_ = 0.80
Within phase errors	*F*_(3, 13)_ = 2.60	*F*_(4.31, 60.34)_ = 2.15	*F*_(12.04, 52.19)_ = 2.06^*^
Total arm visits	*F*_(3, 13)_ = 0.86	*F*_(4.67, 65.44)_ = 1.31	*F*_(12.94, 50.07)_ = 2.31^**^

* *p < 0.05*,

** p = 0.001

α identified as a trend toward significance with a p = 0.067.

**Figure 7:**
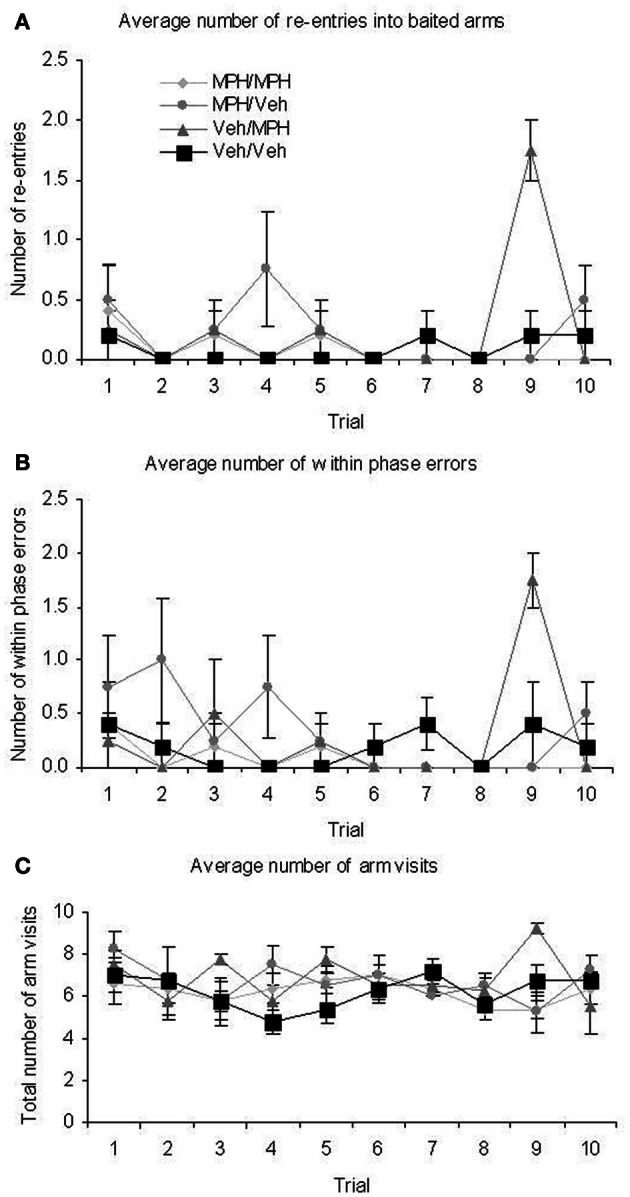
**At the 6 h delay DSWT there were significant interaction effects between trial and treatment on (A) baited re-entry (B) total arm visits (C) within phase errors**. Although interpretation of the interactions displayed in **(A)** and **(C)** is difficult due to the small numbers involved, restricted ANOVA suggested that the interaction they are all due to increased scores recorded for the Veh/MPH group on trial 9. All graphs show mean ± SEM.

There were no significant TRIAL × TREATMENT interactions for latency; mean arm choice time; baited entry; across phase errors or unbaited re-entry. There were significant interactions for baited re-entry; within phase errors and total arm visits (Figure 7). In all cases of significant interactions *post-hoc* ANOVA and tukey analysis revealed a significant difference between the groups on Trial 9. For the baited re-entries and within phase errors the Veh/MPH group differed significantly from all other groups with a higher number of errors. For the total arm visits the same group differed significantly from the MPH/MPH group and the MPH/Veh group. In all cases if this trial was removed from analysis there was no longer a significant interaction. There was no specific outlier which could account for these differences and, although significant, the differences are small and therefore may have arisen through individual differences.

## Discussion

### Effects on non-delayed learning

In the non-delay task behavioral variability (as assessed by standard deviations) decreased across days as would be expected during task learning. This rate of change in variability was the same in both groups at the low dose. At the high dose the vehicle group showed lower variability at the start of testing but both groups reached similar levels within the first three trials and continued at these levels until the end of testing. As is usual with behavioral testing there was a degree of inter and intra-individual variability in performance and this was higher for the time measures (e.g. latency to first arm, mean arm choice time). It is important to note that order effects were controlled for because the order of testing was reversed on alternate testing days and drug administration was evenly distributed across the order of testing. Unfortunately objective statistical analysis of order effects is not possible due to the limited number of animals in each group. Animals were randomly assigned to groups to attempt to control for individual differences but a number of effects arose throughout the results suggesting that management of potential individual differences would have been better achieved using larger group sizes to add power. Unfortunately there are physical constraints on the number of animals that can be feasibly tested in a single day. Thus, it is possible that any lack of effect of MPH reflects the power of our study although we note that our study was able to detect some significant effects of MPH which we discuss below.

Our data revealed that neither dose of MPH had a significant effect on performance of the non-delayed random foraging task compared to the control group, although there was a trend toward a delay in reaching criterion for the higher dose (5.0 mg/kg). This result was unexpected because this task has been shown to rely on transmission between the NAc and hippocampus (Kesner and Dimattia, 1987; Floresco et al., 1997). These two structures would be affected by MPH which acts to increase levels of dopamine and noradrenalin in these structures, respectively. Although we did not directly measure brain activity underlying performance we suggest that it is likely to be changes in NAc dopamine that would affect performance. This is because previous studies have shown that random foraging behaviour, as assessed in the current study in the non-delay task, can be dose-dependently impaired by administration of haloperidol into the NAc shell (Floresco et al., 1996). Furthermore, whilst noradrenalin is known to be a powerful modulator of hippocampal activity (Moore and Bloom, 1979; Loy et al., 1980; Mueller et al., 1982; Madison and Nicoll, 1988; Gereau and Conn, 1994), behavioral studies using radial maze tasks have found noradrenalin manipulations ineffective at altering performance (Myhrer, 2003).

Assuming that it is NAc dopamine that is critical to this task, it is possible that it was the level of training experienced that resulted in our lack of effect of MPH on task performance. The work by Floresco et al. (1996) involved administering a dopaminergic drug to rats which were highly trained and had already reached criterion on the task. However, in the present study, we administered MPH throughout training and therefore it is possible that the effect of increased dopamine is not as evident, if at all, in animals still learning the task. Certainly, learning-dependent effects of NAc dopamine levels have been previously suggested to underlie differences in dopamine effects on other radial maze tasks (Kim and Levin, 1996). A related explanation for the inconsistency may be that the increase in motivation derived from higher accumbens dopamine levels effectively increased distraction (Kim and Levin, 1996) and that, during learning, rather than once trained, this could counter any dopamine-related improvement.

Nonetheless, the training level would not explain why we did not find effects whilst a previous study using orally administered MPH throughout learning did show some effects on a similar task (Zhu et al., 2007). Zhu et al. (2007) examined the effects of orally administered 3.0 mg/kg MPH on non-delay performance when all eight arms of the maze were baited. The results from this study partially concur with our own in that there was no effect on the time taken to reach criterion on the task or locomotor measures. They also found similar main effects of trial on a number of measures, as to be expected with a learning task. However, in contrast to the current results they found some main effects of treatment, which showed improvements in performance with the drug, especially when narrowing to the early phases of learning. There may be a number of reasons for the discrepancy in findings between this study and our own. Firstly, the task employed by Zhu et al. (2007) had all eights arms baited, in contrast to the four baited on our task, making the Zhu et al. (2007) task easier. However, this seems unlikely to explain the difference because the training phase of the DSWT, which is very similar to the task employed by Zhu et al. (2007), also showed no significant effects of treatment. Secondly, although oral administration was employed by Zhu et al. (2007) they used administration via food. This could have altered bioavailability in comparison to our study. It is possible that the changed bioavailability reduced the size and/or duration of the effect of MPH on the relevant neurotransmitters and brain structures. We suggest that this is unlikely because we were using doses and time scales previously validated by blood plasma measures. However, as we did not measure blood or brain levels of the drug in the current study we cannot rule out the lack of effects being due to ineffective doses. Thirdly, Zhu et al. (2007) used younger rats (PND 22-39) with the main effects seen during early phases of testing (PND 22-28), in contrast to our own rats which were PND 60 at the start of testing. We deliberately selected this adult age to mirror the use of cognitive enhancers in adults (Sahakian and Morein-Zamir, 2007) but it is possible that this may have contributed to the differences in findings between the two studies. Certainly, there is evidence that the PFC and, in particular, dopamine innervation of it, does not reach maturity until at least P60 (Kalsbeek et al., 1988), which may affect the neural substrates of the task. It may also mean that performance is generally poorer in younger animals. Indeed, Zhu et al. (2007) suggested that they found effects only at this young age because rats of this age normally perform poorly on the radial maze tasks (Chambers et al., 1996), and show reduced exploratory behaviour (Galef, 1981), effectively creating additional scope for drug effects. Finally, Zhu et al. (2007) conducted their research using both male and female Sprague Dawley rats whilst we used only male Hooded Lister rats in the present study. Although Zhu et al. (2007) report that there were no effects of sex on any of the results, indicating that this is unlikely to account for the differences between the two studies; differences in strain have been shown to affect radial maze performance. For example, it has been demonstrated that Hooded Lister rats performed better than Wistar rats on a radial maze task in the first five trials (Manahan-Vaughan and Schwegler, 2011). In addition, previous work demonstrates that, again in the first five trials on a similar radial maze task, Wistar rats performed better than Sprague Dawley rats (Higashida and Ogawa, 1987). Therefore, the differences seen between the two studies may relate to differing baseline levels of performance in the two strains selected. Based on this previous research, we speculate that Hooded Lister rats may perform better at baseline than other strains and, given the reported baseline-dependency of MPH (Clatworthy et al., 2009) with only poor performers benefitting, this could explain the lack of effect in the current study of a dose previously found to be effective.

Although there was no significant effect of the 5.0 mg/kg MPH relative to the control group beyond a trend toward increasing the time taken to reach criterion, the 5.0 mg/kg group did perform significantly worse than those given 3.0 mg/kg MPH in terms of reaching criterion. Examination of the different measures did not reveal any main effects of treatment over the entire duration or when the analysis was limited to the first five trials. One possible explanation for this is that rats treated with 5.0 mg/kg take longer to reach criterion because they are displaying stereotypic behaviour, confounding performance on the RFNDT. However, this seems unlikely because other radial maze measures were unaffected and locomotor monitoring did not reveal any significant stereotypy in any group. However, it should be acknowledged that the automated recording of stereotypy may be less optimal than direct behavioral observation because the software detects any partial body movement within a defined space. Whilst this will detect stereotypic movements such as head weaving and grooming chains (Taylor et al., 2010), it may also include normal grooming behaviors, which could mask real stereotypic activity. In any event, previous research investigating stereotypy typically uses higher doses of psychostimulants administered intraperitoneally (Walker et al., 2010).

An alternative explanation is that this detrimental effect on task performance at a higher dose is a result of the complex relationship between cognitive function and dopamine levels thought to best describe an inverted *U*-shaped relationship between the two (Roberts et al., 1994; Williams and Goldman-Rakic, 1995; Mattay et al., 2003; Cools and D'Esposito, 2011). Irrespective of the exact neurotransmitter mechanisms, dose-dependent effects of MPH have also been previously reported on other cognitive tasks in both clinical human populations (Sprague and Sleator, 1977; Tannock et al., 1995), and healthy animals (Berridge et al., 2006; Arnsten, 2009; Gamo et al., 2010). However, the prior work in healthy animals, and therefore the most comparable to the present study, has focussed on working memory tasks dependent on the PFC (Berridge et al., 2006; Arnsten, 2009; Gamo et al., 2010). Whilst it is probable that, with the doses employed, there would have been some increase in dopamine and noradrenalin in this region (Berridge et al., 2006) in the present study, we do not think the effects seen were mediated by changes in the PFC because this area is not a critical structure in performance of the RFNDT. Further research would be required to investigate this fully, but to our knowledge this is the first study showing a dose-dependent effect of MPH on a task that is dependent on the hippocampal-NAc circuitry.

### Effects on delayed learning

As for the RFNDT animals were randomly assigned to groups to attempt to control for individual differences but again a number of effects arose throughout the results suggesting that power and therefore sensitivity could have been improved with larger group sizes. Most notably the random allocation to groups unfortunately generated a significant difference in error performance in the MPH/VEH group in the DSWT which we controlled statistically using ANCOVA. However, it is possible that this group effect at 3 h contributed to the lack of significance of the state dependent effect in this group compared to the VEH/MPH group at 6 h (discussed below). In the DSWT we found no effect of MPH in the training phase, which is perhaps unsurprising given its similarity to the RFNDT, albeit even simpler, with only four arms open and all being baited. However, when examining the test phase of the DSWT we did see effects of drug treatment on the number of re-entries into baited arms. The group given MPH during training and test phases did not differ significantly from those given the vehicle in both phases. However, when the drug was only present in one phase there appeared to be an increase in the number of errors relative to both these groups (MPH/MPH and Veh/Veh), although this only reached significance for the Veh/MPH group. Therefore, when the same treatment state, be it MPH or vehicle, was present in both the training and test phase there was no overall difference in performance between drug treated animals and controls. In contrast when the treatment state in the phases differed, performance may be impaired in drug treated animals. This represents a state-dependent effect similar to state-dependent learning. A number of studies have previously reported state-dependent learning with MPH in humans (Swanson and Kinsbourne, 1976; Shea, 1982; Pozzi and Hartley, 1984) but they have all focused on a clinical population with ADHD or hyperactivity. Moreover, even within this population results are conflicting with Becker-Mattes et al. (1985) failing to find the effect. Therefore, to our knowledge, this is the first report indicative of a state-dependent effect in healthy individuals given MPH. Although a state dependent learning effect would be likely to manifest in the across phase errors this measure has reduced sensitivity as it has a ceiling score of four. In addition it is interesting that the state dependent effect occurs in the re-entries to baited arms in the test phase which suggests that animals have difficulty matching location to reward when the contextual state is different between phases.

Although the state dependent effect observed may relate to changes in arousal level, such as through noradrenergic changes, or motivation, this seems unlikely in the current study because there were no group effects on the number of pellets consumed, with all rats consuming the maximum. Moreover there were no differences in the latency of arm choices, which may be expected to differ if motivation or arousal were affected. Therefore, it is possible that the state dependent effect we observed is due to specific actions of the drug on neurotransmitter levels. Previous work has shown that dopamine in the PFC is important in performance on the DSWT (Floresco and Phillips, 2001). Microdialysis has shown that there is an increase in dopamine efflux during the DSWT training phase which is associated with searching and eating the reward pellets (Phillips et al., 2004). This increase then returns to baseline levels after approximately 5 min and it is thought that the hippocampus is responsible for maintaining the memory for food reward location during the delay. However, during the test phase, Phillips et al. (2004) report an almost identical increase in dopamine efflux that is present even when the food reward is unavailable. When the delay is unpredictably increased (to 1 or 6 h) the level of dopamine efflux decreases and the number of errors made increases during the test phase. In the current study we would expect the normal efflux of dopamine to have occurred during the training phase. However, although we increased the delay to 6 h this was from only 3 h, unlike Phillips et al. (2004) who increased it from 30 min to 6 h. As such, we suggest that some dopamine efflux would still have occurred at the 6 h delay, comparable to that found by Phillips et al. (2004) for their 1 h delay as the magnitude of increase (i.e., multiplied by two) was the same. However, since we did not measure dopamine levels directly this remains an assumption.

In sum our results could have implications for individuals attempting to achieve cognitive enhancement with MPH. From the current study it seems that at best performance may remain unchanged at the doses employed and at worst performance could be impaired. When delays are involved and MPH is taken in both phases, there is no improvement in performance compared to not having MPH at all and, at worse, when only taken in one phase, it may actually be detrimental. A similar situation in humans could amount to taking MPH during revision and during an exam (with this having no effect on performance), whilst only taking the drug at one stage (revision, or exam only) might actually decrease performance. Our findings, in rats on the radial maze at least, suggest that MPH in healthy rats is not an effective cognitive enhancer, although there are admittedly some important differences between animals working for an immediate food reward on a maze and students working toward the delayed goal of passing an exam. Although we have noted some limitations to our study in terms of power and sensitivity our findings are supported by a recent review (Lynch et al., 2011). Of course, the functions assessed in the present study were limited to specific measures relating to goal-directed learning and memory and therefore one could argue that there are many other functions which could be enhanced. However, it should be noted that these functions were deliberately chosen because of their likely sensitivity to the neurochemical effects of MPH and therefore the negative findings of this study are of interest.

### Conflict of interest statement

The authors declare that the research was conducted in the absence of any commercial or financial relationships that could be construed as a potential conflict of interest.
